# Effects of Peer-Supported and Self-Guided Exercise on Self-Reported Anxiety and Depression among Young Adults—A Pilot Study

**DOI:** 10.3390/jfmk8030125

**Published:** 2023-09-01

**Authors:** Xihe Zhu, Michael D. Kostick, Justin A. Haegele

**Affiliations:** Department of Human Movement Sciences, Old Dominion University, Norfolk, VA 23529, USAjhaegele@odu.edu (J.A.H.)

**Keywords:** college student, decay, exercise intervention, mental health, physical activity

## Abstract

Mental health conditions such as anxiety and depression became heightened issues for college-aged young adults during the global pandemic. The main purpose of this study was to examine the effects of a peer-supported exercise intervention on young adults (vs. self-guided exercise) who reported elevated levels of anxiety and/or depression. A parallel group design was used where young adults (*n* = 27) were randomly assigned to either a peer-supported or self-guided exercise group which lasted for eight weeks. The generalized anxiety and depression subscales of the Counseling Center Assessment of Psychological Symptoms (CCAPS-34) were measured for a baseline and then at 4-week, 8-week, and 12-week follow-up. Analyses of covariance (ANCOVA) with repetitive measures show that peer-supported and self-guided exercise programs reduced participant anxiety and depression scores; however, intervention decay for the peer-supported exercise intervention was more severe than that for the self-guided group. Self-guided exercise had a longer-lasting effect than the peer-supported alternative and could be a cost-effective approach to combat anxiety and depression issues among young adults.

## 1. Introduction

Over the past several years, the COVID-19 pandemic has brought mental health issues such as anxiety and depression into the international spotlight [1,2]. While anxiety is a normal occurrence throughout human interactions with the environment, events, and other individuals, individuals with anxiety disorders tend to experience persistently excessive or intense worries that negatively affect their everyday lives [3]. On the other hand, depression is characterized by disordered feelings of sadness and a decrease in interest towards regularly enjoyed activities in a persistent manner [4]. While anxiety and depression rates have risen overall during the COVID-19 pandemic [1], some populations are more susceptible to increases in mental health issue prevalence. For example, college-aged young adults have been specifically identified as having increased rates of anxiety and alarming rates of moderate-to-severe-level depression before [5] and in the wake of the public-health-mandated quarantines associated with COVID-19 [6]. Therefore, remediating public health attention and resources must be directed toward reducing anxiety and depression among this vulnerable population. 

Contemporary research has sought to quantify the mental health benefits of physical activity participation [7]. Physical activity participation has been documented as a preventative and remedial measure for mental health issues [8,9]. For example, observational studies have shown a positive association between physical activity participation and a lower prevalence of anxiety and depression [10], taking part in high-intensity physical activity decreases the risk of a diagnosis of depression [11], and that engagement in cardiovascular and muscle strengthening physical activities could be used as a proactive practice in precluding the ill effects of stress and anxiety [8]. Supporting this, some mental health specialists have even indicated that physical activity guidelines should be updated to reflect engagement in enjoyable, leisure-time physical activity rather than forced incidental movement, proposing that mental and physical health could be prioritized together through physical activity [12,13,14]. 

When used as a prescription or treatment, moderate-to-vigorous physical activity has been shown to have positive outcome effects for individuals experiencing depression, schizophrenia, and bipolar disorders [15]. For example, using a randomized control trial, Borrega-Mouquinho and associates [16] observed that young adult participants in a high-intensity interval training intervention self-reported a significant decrease in symptoms of anxiety (z = −2.449, *p* = 0.014, ES = 0.408), depression (z = −4.161, *p* < 0.001, ES = 0.693), and stress (z = −4.184, *p* < 0.001, ES = 0.697) after the completion of the program. Mental-health-related benefits have also been explored among college student populations specifically. For example, Herbert and her colleagues [17], in randomized control trial pilot studies on college students (*n* = 19), observed that students placed in an online physical activity intervention group reported decreased levels of depressive symptoms after completion of the intervention (t(18) = 3.38, *p* < 0.005, d = 0.62). 

While it appears clear that physical activity engagement can help to reduce rates of anxiety and depression among young adults and college-aged students, physical activity levels among this population have decreased over the past few years, most notably during the COVID-19 pandemic [18]. Therefore, there is a clear and urgent need to identify mechanisms to promote physical activity, and therefore reduce anxiety and depression rates, among this population. Given that peer-supported exercise programs may simultaneously enhance physical activity engagement while also providing mental-health-related benefits, in this study, we evaluated the mental health benefits of a peer-supported exercise intervention for young adults. 

Peer support can not only serve as a referral source but has been shown to be an effective tool for reducing depressive symptoms among multiple populations, including college students [19,20,21]. For example, in a study evaluating the relationship between group exercise membership and general state anxiety among college students, Patterson and colleagues [22] found group exercise membership to be significantly related to lower anxiety scores. Villatte and colleagues [23] provide a thorough description of family-based, personal, social, and academic correlates to depression and anxiety among young adults. Complementing this research, Blanco and colleagues [24] identified that students with relationship-related stressors, as well as those with low social support, are among the most likely to experience poor mental health. Therefore, an important element of potential treatment programs for mental health may be peer support. The association between group (i.e., peer support) settings and improved health is largely supported by research highlighting the importance of social connection to one’s overall well-being [22]. Thus, an exercise invention with peer support may yield greater benefits for those at risk or experiencing elevated levels of anxiety or depression. 

The COVID-19 pandemic, and associated reductions in physical activity and increased rates of anxiety and depression, call for the examination of innovative exercise programs that can be effectively conducted in virtual contexts. The main purpose of this study was to examine the effects of a peer-supported exercise intervention on young adults (vs. self-guided exercise) who had elevated levels of anxiety and/or depression. These young adults were screened with mild or moderate anxiety or depression but were not clinically diagnosed with severe disorders and were not concurrently receiving psychotherapy or antidepressants for treatment. The secondary purpose of the study was to examine the decay of intervention impact through a post-intervention follow-up, a practice that has been sparsely reported in the literature [25].

## 2. Methods

### 2.1. Participants and Research Design

A parallel group design was used where the participants were randomly assigned to either a peer-supported or self-guided exercise group. Participants for the study were recruited from the campus of a large research institution during the Fall 2021 semester. Interested prospective participants met virtually with a member of the research team to discuss the study and gain informed consent. Once consent was obtained, prospective participants were asked to complete a health-screening questionnaire to assess eligibility for participation in the research study. In order to be included as a participant in the study, individuals needed to (a) be 18–26 years of age, (b) self-report elevated anxiety and/or depression levels above the Center for Collegiate Mental Health (CCMH) [26] cutoffs: 65% percentile rank in generalized anxiety and 59% percentile rank in depression (details described in Section 2.3), (c) have no cardiac or other health conditions that could medically prevent them from engaging in moderate-to-vigorous physical activity, (d) not be clinically diagnosed with anxiety/depression or taking antidepressant medication or receiving other clinical/psychotherapy treatments, and (e) be willing to complete all of the data collection requirements and exercise program.

**Figure 1:**
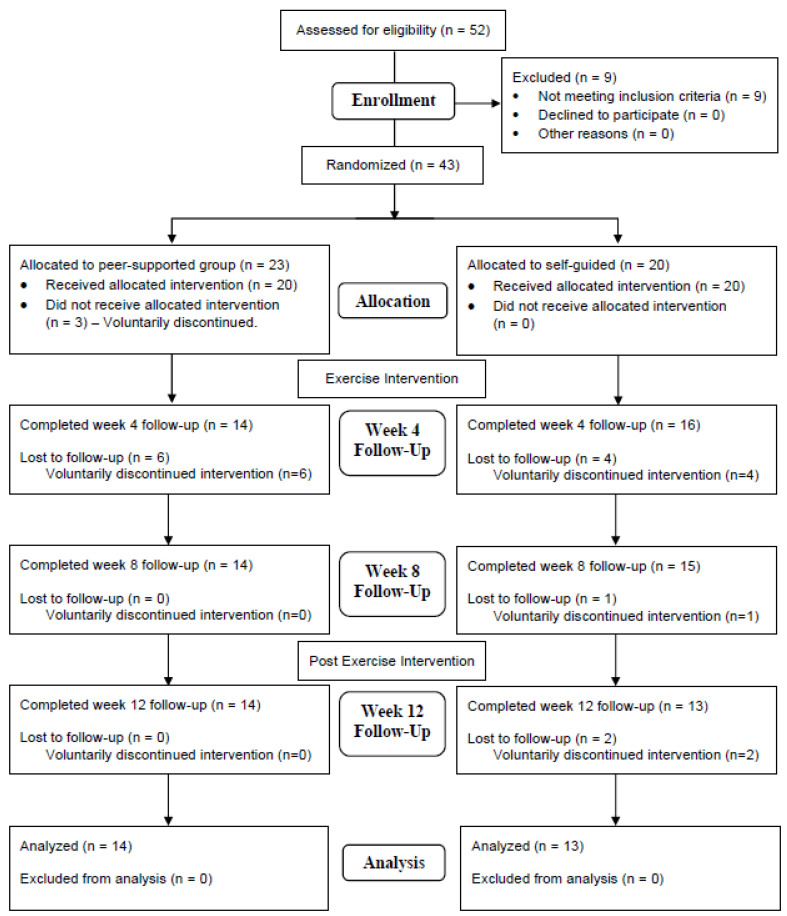
Study flowchart.

Counting the screening questionnaire, we had a total of four waves of data collection on participant anxiety/depression levels, with an interval of four weeks, including two during and immediately after the intervention, and the last one, four weeks after the study. Each participant received Amazon.com gift cards totaling up to USD 200 as an incentive for completing all waves of data collection. The flowchart of participants (Figure 1) depicts the recruitment and retention of participants throughout the process of the research study. The study protocols were reviewed and approved by the researchers’ institutional review board.

Of the 52 prospective participants who gave informed consent, 9 did not meet one or more of the inclusion criteria. At the beginning of the enrollment process, 43 participants were randomly allocated into one of two groups: (1) an online peer-supported exercise group or (2) a self-guided exercise group. As seen in the study flowchart, Figure 1, the peer-supported exercise group began with 20 participants and met over Zoom two to three times per week for a total of 150 min per week during an 8-week timeframe. These participants completed a high-intensity interval training (HIIT) exercise program led by two different members of the research team. The self-guided exercise group began with 20 participants who were tasked with completing the same HIIT exercise program on their own, yet they were still asked to engage in the exercise program for 150 min per week for the 8-week timeframe. By the week-four follow-up survey, six participants had voluntarily discontinued their participation in the peer-supported exercise group while four participants had voluntarily ended their participation in the self-guided group.

### 2.2. Peer-Supported and Self-Guided Exercise

In order to obtain implementation fidelity, participants from both peer-supported and self-guided groups were tasked with completing an identical eight-week exercise program consisting of dynamic warmups, high-intensity interval training (HIIT) exercise bouts, and a stretching cooldown. A HIIT exercise routine was selected as research has indicated cardiovascular interval training may maximize overall health-related fitness outcomes [27]. Each week, participants in the self-guided group received an email prompt from the research team with the HIIT routine for that respective week within the program (e.g., “Week 1” exercise program on the first week). Self-guided group participants were asked to respond to the email correspondence in order for the research team to determine continuance intentions. Peer-supported group participants met with their designated exercise peer supporter through Zoom where attendance was taken using their anonymous, randomized participant identification number. Within the Zoom session, a member of the research team provided motivational support verbally (e.g., “keep going!” or “good job!”) and guided all physical activities in the same order as participants in the self-guided exercise groups to maintain consistency of exercise program adherence between groups. The research team, cognizant of academic responsibilities and potential time conflicts, offered two separate peer-supported exercise group opportunities: two days per week for 75 min each session or three days per week for 50 min each session.

A member of the research team compiled bodyweight isotonic and isometric exercises that would target upper-body, core, and lower-body muscle groups. Dynamic warmups each week included exercises such as arm circles, alternating toe touches, butt kicks, touchdown jacks, jumping jacks, and high knees. Warmup exercises would be completed in 30 s intervals with a 30 s rest between exercises. Typically, participants completed three to four warmup exercises twice in a cyclical manner. In between the warmup and the circuit, participants were given a one-minute rest period.

Throughout the circuit portion of the exercise routine, participants were tasked with completing two to three exercises in 30 s bouts consecutively followed by a 30 s rest period. Lower-body exercises included squats, alternating side lunges, glute bridges, and others. Core exercises included side crunches, mountain climbers, plank sidewalks, and others. Upper-body exercises included pushups, overhead punches, arm circles, and so forth. Weekly circuits primarily consisted of four to five high-intensity bouts containing three exercises each and 30 s rest periods in between each group. The circuit would be completed four (three days per week group) or seven (two days per week group) times with 60 s rest periods between circuits.

Cooldown exercises were consistent throughout the entirety of the eight-week program. Exercises targeted flexibility and consisted of baby cobra, downward dog, rag doll, triangle pose stretch, and others. Stretches were done contiguously, with each stretch held for 30 s.

### 2.3. Instrumentation

To accommodate the repetitive-measure design, we used the generalized anxiety and depression subscales of the Counseling Center Assessment of Psychological Symptoms (CCAPS-34) [26], which were designed for brief and repeated measurements for college students [28]. Specifically, there are six items each for generalized anxiety and depression subscales. Each item has a short statement that is anchored with a five-point scale, ranging from 0 = “Not at all like me” to 4 = “Extremely like me”. For example, an item for generalized anxiety reads “I am anxious that I might have a panic attack in public”, and an item for depression states “I feel sad all the time.” The internal consistency for the college students was α = 0.83 for generalized anxiety and α = 0.88 for depression subscales. The current norm was established based on 59,606 students seeking counseling at 120 colleges/universities during the 2011–2012 academic year, with the elevated cutoffs for generalized anxiety raw score = 2.10 (65% percentile rank) and depression raw score = 1.75 (59% percentile rank) [26]. Since this project was situated at a participating institution of the CCMH initiative, we used these cutoffs as one of the screening criteria.

### 2.4. Statistical Analysis

For the purposes of this study, we conducted descriptive statistics and inferential statistical analyses. Specifically, we conducted frequency analysis on participant demographic variables such as gender and ethnicity and computed the mean and standard deviations of the participant age. In addition, we checked the central tendency, distribution, and variability assumptions for parametric analyses. To examine the depression and generalized anxiety changes for peer-supported and self-guided exercise groups, we ran analyses of covariance (ANCOVA) with repetitive measures where the baseline, 4-week, 8-week, and 12-week follow-up scores were recorded. Considering the moderate correlation between the depression and generalized anxiety baseline scores (r = 0.53, *p* < 0.01), we used the baseline depression score as the covariate for anxiety analysis, and vice versa. Through these analyses, we examined the within-subject (measures), between-subject (peer-supported vs. self-guided) and measures×group interaction effects. The analyses were conducted using SPSS (ver. 27; IBM, Amour, NY, USA), and α = 0.05 was kept as the threshold for statistical significance tests.

## 3. Results

The participants were similarly aged for both peer-supported (*n* = 14) and self-guided (*n* = 13) exercise groups. As seen in Table 1, most participants were undergraduate students, with four graduate students in both groups. Most participants identified as female, with only one male in the peer-supported group and one selecting “other” when reporting gender in each exercise group. The participants were ethnically diverse, with details shown in Table 1. The participant depression and generalized anxiety subscale scores are presented in Table 2, with similar baseline mean scores across groups, and these scores were higher than the elevated cutoffs for both depression = 2.10 and generalized anxiety = 1.75 [26].

Overall, there was no significant between-subject effect for depression (*F*_1,24_ = 2.09, *η*^2^ = 0.08, *p* = 0.16) or generalized anxiety (*F*_1,24_ = 0.48, *η*^2^ = 0.02, *p* = 0.49), suggesting no significant overall difference in group marginal means for these measures between the peer-supported and self-guided exercise groups. The ANCOVA results showed a significant within-subject effect in generalized anxiety (*F*_3,22_ = 4.69, Pillai’s λ = 0.39, *η*^2^ = 0.39, *p* = 0.01), but a non-significant within-group effect in depression (*F*_3,22_ = 0.05, Pillai’s λ = 0.01, *η*^2^ = 0.01, *p* = 0.98), suggesting that while participant generalized anxiety significantly declined during the study period, depression did not. These findings showed that both peer-supported and self-guided exercises were able to significantly reduce participant generalized anxiety and that there was a decline in participant depression, although it was not statistically significant.

**Figure 2:**
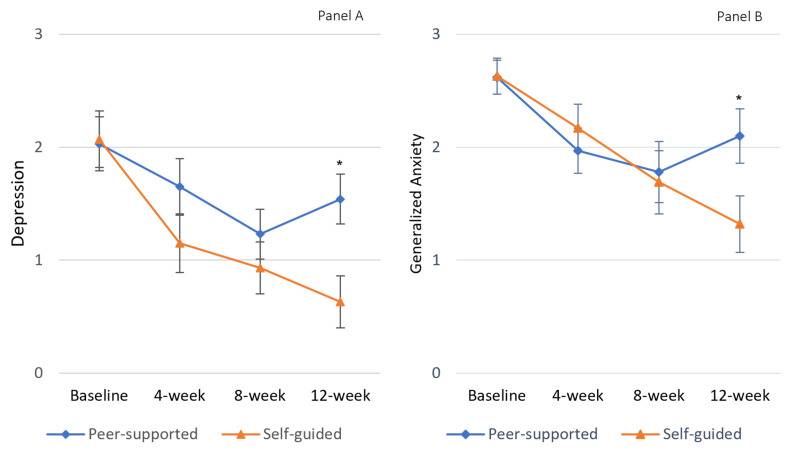
Estimated marginal means for generalized anxiety (Panel **A**) and depression (Panel **B**) subscale scores. Note: * *p* < 0.05 adjusted for participants’ baseline depression for repetitive measures of generalized anxiety, and vice versa.

As displayed in Table 2, there were significant measure×group effects for both generalized anxiety (*F*_3,22_ = 3.23, Pillai’s λ = 0.39, *η*^2^ = 0.12, *p* = 0.03) and depression (*F*_3,22_ = 3.91, Pillai’s λ = 0.27, *p* = 0.98, *η*^2^ = 0.14, *p* = 0.01), showing a significant group effect on the repetitive measures. As seen in Figure 2B, upon adjustment of participant baseline anxiety scores, the participant depression scores steadily declined for the self-supported group with peak-to-trough variation Δ = 1.29, whereas the trough emerged immediately after the exercise intervention for the peer-supported group Δ = 0.85 (Table 2). A similar pattern emerged for the depression scores as well (Figure 2A); that is, the participant depression scores steadily declined for the self-supported group with peak-to-trough variation Δ = 1.45, while the trough formed immediately after the exercise intervention for the peer-supported group Δ = 0.78 (Table 2). Pairwise comparison showed significant differences in participant depression and generalized anxiety scores at 12-week follow-up measure while no significant difference existed at baseline, 4-week, or 8-week measures between peer-supported and self-guided exercise groups (Figure 2), suggesting that the self-guided exercise may have a longer-lasting effect than the peer-supported one, with little or no decay four weeks after the study.

## 4. Discussion

The main purpose of this study was to examine the effects of a peer-supported exercise intervention on young adults (vs. self-guided exercise) who had elevated levels of anxiety and depression. In our study, participants in each intervention group, regardless of delivery modality, experienced reduced anxiety and depression scores after the completion of the exercise intervention. This finding is important, as it supports the role that exercise programs can play in reducing mental health issues even when delivered in online, socially distant formats [29]. Of further note, our study included college-aged students who had previously experienced moderate-to-high levels of general anxiety and/or depression. Therefore, the findings here have further value in supporting the effectiveness of online-delivered exercise programs for an already vulnerable population. These findings are clearly well aligned with what was seen by Borrega-Mouquinho and colleagues [16], who identified positive changes in anxiety and depression symptoms among college-aged students who had previously experienced moderate-to-high levels of general anxiety and/or depression, and suggest that perhaps programs like this should be adopted on college campuses and by offices of health management when exploring options to help serve those experiencing mental health issues.

Interestingly, the self-guided exercise group in our study appeared to be just as effective in reducing anxiety and depression as the peer-supported group. This result was surprising, given that there is a substantial body of literature that supports the relationship between the availability of social support and reduced anxiety and depression symptoms among college students [19,20,21,22]. There are a number of potential explanations for this particular finding. For example, our participants were not permitted to select whom they received peer support from and were rather assigned to a particular person or group. Therefore, it is possible that while our participants received an intervention intended to provide social support in the form of peer support, they may not have experienced what they subjectively perceived as social or peer support [30]. Therefore, the prescribed peer support may not have had the intended impact, and participants may not have enjoyed the additional mental health benefits associated with peer support [23], which potentially explains similar results between intervention groups. This result, though, also has important practical implications. That is, perhaps the lack of difference between these two groups demonstrates that self-guided programs can be equally effective as peer-supported programs while being more cost- and time-efficient because of the lack of necessity for the involvement of the peer supporter. More research should be conducted to further explore this, but this finding could support the implementation of more cost-effective programs (self-guided) to combat anxiety and depression increases among college students with elevated but below clinical threshold levels of generalized anxiety and depression.

The secondary purpose of this study was to examine the decay of intervention impact through post-intervention follow-up. Interestingly, while differences did not emerge between groups with regard to the intervention effects, there were significant differences between the self-guided group and peer-supported group at the 12-week follow-up. That is, it appears that the decay effect for the peer-supported group was evident four weeks immediately after the post-intervention data collection, where participant depression and generalized anxiety scores moved higher. As seen in Figure 2, this was not the case for the self-guided group. These findings suggest that there was a prolonged effect for the self-guided exercise group, whereas the effect associated with the peer-supported group discontinued when the intervention was completed. This is an important finding, again suggesting the utility of self-guided exercise groups, while perhaps also suggesting that the peer-supported group’s effect may have been buoyed by the social interactions associated with participation in the program, rather than the exercises that participants engaged in. Decay effects are also evident in short-duration physical activity or exercise programs, where some suggest that intervention momentum may be compromised because of the relatively short runway for gains during short programs [31]; however, if this were the cause in this particular study, it should influence both intervention groups similarly given they experienced the same exercise programs of the same duration.

We computed the peak-to-trough variation within the exercise groups. For peer-supported exercise groups, the trough seemed to form immediately at the end of the 8-week intervention, whereas as seen in Table 2, the trough was found at the 12-week follow-up for the self-guided intervention. Considering that we had only followed the participants for this limited 12-week period, it is likely that the trough levels would differ if a longer period were followed for these participants, which may warrant further study. Nevertheless, the larger peak–trough variation as seen in Table 2 and Figure 2 was clearly favorable for the self-guided exercise group.

Limitations of the study did exist. First, while we were able to test the significant differences, the sample size is relatively small. While a decline in anxiety and depressive symptoms was seen throughout the study, academic variables might have had an effect on participant reports. By the 8-week follow-up surveys, the academic semesters for participants were coming to a close as most participants had already finished the majority of their final examinations and projects by that point. Researchers conducting future studies using college-aged young adults may want to consider scheduling their exercise programs around the academic calendar. It should be noted that the anxiety and depression symptom scores were self-reported; while commonly used in screening and research practices, the self-reported symptoms may need to be validated against clinical interviews and/or diagnoses [32].

In summary, we examined the effects of a peer-supported exercise intervention on young adults who had elevated levels of anxiety and/or depression. Our data showed that the peer-supported exercise intervention had a positive effect on anxiety and depression symptoms. However, these effects were similar to those experienced by participants in a self-guided exercise group. In addition, the intervention decay for the peer-supported exercise intervention was greater than that for the self-guided group. Therefore, while our study supports the intervention effects of exercise interventions in reducing mental health issues, the findings do not favor peer-supported experiences over those that are self-guided.

## Figures and Tables

**Table 1:** Participant demographic characteristics of peer-supported and self-guided groups.

Variable	Peer-Support Group	Self-Guided Group
Age (Yr)	21.71 ± 2.37	22.15 ± 2.48
Range	[18, 26]	[19, 26]
College education (*n*)		
Undergraduate	10	9
Graduate	4	4
Gender (*n*)		
Female	12	12
Male	1	0
Other	1	1
Ethnicity (*n*)		
Asian	1	1
Black	6	5
Latino	2	1
White	4	5
Other	1	1

**Table 2:** Generalized anxiety and depression scores for peer-supported and self-guided exercise groups.

Subscale	*F*_3,22_	*η* ^2^	*p* *	Group	Measure	Score	Δ ^†^
Generalized Anxiety	3.23	0.12	0.03	Peer-supported	Baseline	2.61 ± 0.61	0.85
					4-week	1.95 ± 0.99	
					8-week	1.76 ± 1.04	
					12-week	2.08 ± 0.98	
				Self-guided	Baseline	2.64 ± 0.71	1.29
					4-week	2.19 ± 0.83	
					8-week	1.71 ± 1.11	
					12-week	1.35 ± 1.09	
Depression	3.91	0.14	0.01	Peer-supported	Baseline	2.01 ± 1.10	0.78
					4-week	1.64 ± 1.05	
					8-week	1.23 ± 0.76	
					12-week	1.54 ± 0.87	
				Self-guided	Baseline	2.09 ± 0.99	1.45
					4-week	1.17 ± 0.99	
					8-week	0.94 ± 0.95	
					12-week	0.64 ± 0.93	

Note: * Adjust for participants’ baseline depression for repetitive measures of generalized anxiety, and vice versa. ^†^ Peak-to-trough variation within the exercise groups.

## Data Availability

The raw data are unavailable due to privacy or ethical restrictions. The aggregated data are available through the corresponding author.
